# Intraspecific phenotypic variation in a fish predator affects multitrophic lake metacommunity structure

**DOI:** 10.1002/ece3.878

**Published:** 2013-11-14

**Authors:** Jennifer G Howeth, Jerome J Weis, Jakob Brodersen, Elizabeth C Hatton, David M Post

**Affiliations:** 1 Department of Ecology and Evolutionary Biology, Yale UniversityNew Haven, Connecticut, 06520-8105; 2 Department of Biological Sciences, University of AlabamaTuscaloosa, Alabama, 35487-0206; 3 Department of Fish Ecology and Evolution, EAWAG Swiss Federal Institute of Aquatic Science and Technology, Centre of Ecology, Evolution, and BiochemistryKastanienbaum, Switzerland

**Keywords:** *Alosa pseudoharengus*, phytoplankton, size-selective predation, spatial mosaic, zooplankton

## Abstract

Contemporary insights from evolutionary ecology suggest that population divergence in ecologically important traits within predators can generate diversifying ecological selection on local community structure. Many studies acknowledging these effects of intraspecific variation assume that local populations are situated in communities that are unconnected to similar communities within a shared region. Recent work from metacommunity ecology suggests that species dispersal among communities can also influence species diversity and composition but can depend upon the relative importance of the local environment. Here, we study the relative effects of intraspecific phenotypic variation in a fish predator and spatial processes related to plankton species dispersal on multitrophic lake plankton metacommunity structure. Intraspecific diversification in foraging traits and residence time of the planktivorous fish alewife (*Alosa pseudoharengus*) among coastal lakes yields lake metacommunities supporting three lake types which differ in the phenotype and incidence of alewife: lakes with anadromous, landlocked, or no alewives. In coastal lakes, plankton community composition was attributed to dispersal versus local environmental predictors, including intraspecific variation in alewives. Local and beta diversity of zooplankton and phytoplankton was additionally measured in response to intraspecific variation in alewives. Zooplankton communities were structured by species sorting, with a strong influence of intraspecific variation in *A. pseudoharengus*. Intraspecific variation altered zooplankton species richness and beta diversity, where lake communities with landlocked alewives exhibited intermediate richness between lakes with anadromous alewives and without alewives, and greater community similarity. Phytoplankton diversity, in contrast, was highest in lakes with landlocked alewives. The results indicate that plankton dispersal in the region supplied a migrant pool that was strongly structured by intraspecific variation in alewives. This is one of the first studies to demonstrate that intraspecific phenotypic variation in a predator can maintain contrasting patterns of multitrophic diversity in metacommunities.

## Introduction

Across a heterogeneous landscape, spatially variable ecological selection can exert differential local selection pressures on phenotypes among populations (Thompson 2005; Chaves-Campos et al. 2011) and can lead to population-level divergence in ecologically important traits within species (Losos 1994; Benkman 1999). Recent research suggests that this population differentiation, or intraspecific variation, can influence the probability of speciation and generate diversifying ecological selection on local community structure (Whitham et al. 2003; Palkovacs and Post 2009; Pantel et al. [Bibr b41][Bibr b42]). Notably, contemporary studies of ecological diversification in vertebrate predators indicate that population-level divergence in foraging traits and behavior (e.g., Palkovacs and Post 2008) can dramatically alter the community composition of lower trophic levels and influence ecosystem function (Post et al. 2008; Harmon et al. 2009; Bassar et al. 2010). Much of this work on the ecological effects of diversification within predators assumes that local populations are situated in communities that are unconnected to similar communities within a shared geographical region. Yet insights from metacommunity ecology suggest that the dispersal of species from the lower trophic levels among communities in the landscape may also be important for species diversity and composition in response to intraspecific ecological diversification within predators (Howeth and Leibold 2010a).

An emerging spatially explicit framework of evolutionary ecology, metacommunity theory, suggests that the degree of ecological diversification within a species and its consequent impacts on the community should in part depend upon the level of population and community connectivity in the landscape (Urban et al. 2008). Metacommunity ecology emphasizes the interaction of species dispersal rates among communities and the local environment in structuring species diversity at multiple spatial scales (Leibold et al. 2004; Cadotte 2006). Although no studies to date have addressed the functional role of intraspecific variation in predators within metacommunities, previous research evaluating the influence of predators in landscapes suggests that acknowledging community connectivity may confer additional insights into effects of their diversification. For example, recent empirical work indicates that the ecological role of predators in communities in part depends upon the degree of predator foraging selectivity relative to prey dispersal rates in the landscape (Howeth and Leibold 2010a). In contexts where predators are selective and prey dispersal rates are low to moderate, predators can exert strong selection on prey communities leading to low levels of spatial community turnover (beta diversity) via species sorting (Chase et al. 2009). Selective predators can also exclude preferred prey or reduce the abundance of dominant competitors, thereby altering local and regional species richness (Paine 1966; Kneitel and Miller 2003). If prey dispersal rates in the region are high, however, the prey dispersal may homogenize local community composition via mass effects (source-sink dynamics) regardless of differences in predator identity or foraging traits (Holyoak and Lawler 1996; Howeth and Leibold 2010a).

Intraspecific phenotypic variation in a predator in a landscape may yield a spatial mosaic of ecological selection and opportunities for differential local sorting to affect multiscale species diversity in metacommunities. Opportunities for sorting will depend upon the strength of prey dispersal in the region relative to functional differences in predation pressure and other environmental processes. Additionally, the relative influence of dispersal and predation in shaping metacommunity structure may vary by trophic level through direct or indirect predator effects (Howeth and Leibold 2008; Chase et al. 2010) and differences in dispersal ability of component taxa (Beisner et al. 2006). The planktivorous fish alewife (*Alosa pseudoharengus*) and its freshwater habitat of the New England coastal lake landscape serve as a model system in which to study intraspecific phenotypic variation in multitrophic metacommunities. Alewives have spatially diversified into two life-history types: the ancestral anadromous form which resides in the ocean but spawns in coastal lakes and the derived landlocked form which inhabits lakes year-round (Palkovacs et al. 2008). The presence and intraspecific diversification of alewife yield coastal lake metacommunities supporting three food web configurations: lakes with anadromous, landlocked, or no alewives (Post et al. 2008). The alewife is an archetypal size-selective predator that preys upon large-bodied zooplankton, including *Daphnia,* in lake communities (Brooks and Dodson 1965; Post et al. 2008). Landlocked alewives have diverged in morphological foraging traits from anadromous alewives (Palkovacs and Post 2008) which facilitates selection of smaller-bodied zooplankton prey (Palkovacs and Post 2009). Recent experimental work demonstrates that this divergence in prey selectivity differentially alters zooplankton species diversity between the two life-history forms of alewife and relative to lakes without alewife (Palkovacs and Post 2009). The differential prey selectivity, together with differences in lake residency of the two life-history forms of alewife, additionally yields contrasting zooplankton community structure in the three lake types and affects the strength of trophic cascades in natural lake ecosystems (Post et al. 2008). Large-bodied zooplankton occur year-round in lakes without alewives, are extirpated or rare in the presence of anadromous alewives (summer), and remain at low densities in landlocked lakes (Post et al. 2008). The rarity of large-bodied zooplankton in anadromous lakes results in increased edible phytoplankton biomass relative to landlocked and no alewife lakes (Post et al. 2008). This evidence for differential trophic interactions by lake type also suggests possible consequences for species diversity in multiple trophic levels.

In this study, we test the relative response of multitrophic plankton metacommunity structure to intraspecific variation in a predator and spatial processes related to species dispersal in coastal lakes that contain anadromous alewives, landlocked alewives, and no alewives. First, we determined whether zooplankton and phytoplankton composition was more strongly influenced by spatial or local environmental predictors, including predation from alewives. We hypothesized that local environmental structuring of zooplankton and phytoplankton in the absence of spatial signatures suggests species sorting in the lake metacommunity, where plankton dispersal is high enough for species to arrive to each community and species subsequently sort through the local environmental filter (Leibold et al. 2004; Cottenie 2005). Alternatively, joint control of plankton community structure by environmental and spatial processes reflects dispersal limitation in the plankton (e.g., species range boundaries, Leibold et al. 2010) or mass effects in the plankton metacommunity (Cottenie 2005). Second, we predicted that multitrophic species diversity and composition would respond to intraspecific variation in alewives and thus measured the response of zooplankton, phytoplankton, and their functional groups defined by size-based edibility to the three food web configurations represented in the coastal lake metacommunity.

## Materials and Methods

### Study system

**Figure 1:**
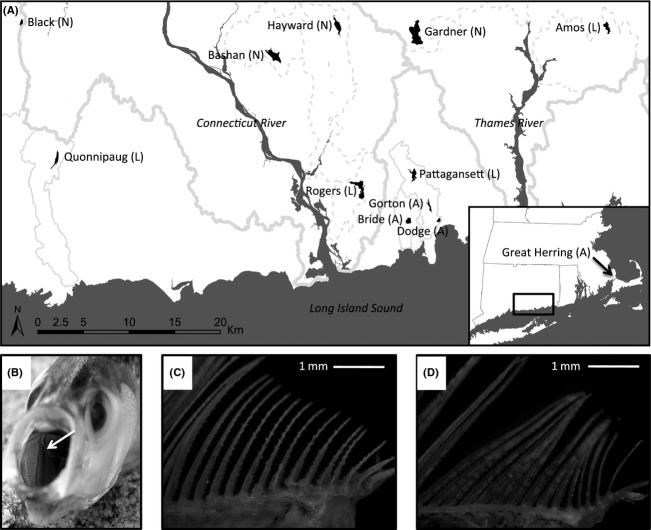
(A) Spatial location of the 12 study lakes in Connecticut and Massachusetts (inset), USA. Lakes are labeled as having anadromous (A), landlocked (L), or no alewives (N), *Alosa pseudoharengus*. For the 11 Connecticut lakes, thick gray lines show the boundaries of the two major regional watersheds in the study region: the Connecticut River watershed, and the Thames River watershed. Within those two major watersheds, dotted gray lines denote the regional watersheds of each of the study lakes. Between the two major watersheds, thin solid gray lines show the boundaries of the smaller coastal watersheds. (B) Anterior view of gill rakers (arrow) in an alewife. The two life-history forms of alewife have diverged in foraging traits, including gill raker spacing: (C) anadromous gill rakers and (D) relatively narrow landlocked gill rakers. Photo credits: (B) D. Post and (C, D) E. Palkovacs from Post et al. (2008).

We sampled zooplankton and phytoplankton in 12 lakes in Connecticut and Massachusetts, USA (Fig. 1A; Table S1). There were four replicate lakes representing each of the three lake types defined by the presence and life-history form of alewives (*Alosa pseudoharengus*): anadromous alewife, landlocked alewife, or no alewife lakes. In Connecticut, only three anadromous populations have access to lake spawning habitat (Palkovacs et al. 2008). As a consequence, an anadromous lake was sampled on Cape Cod, Massachusetts. The 11 coastal Connecticut lakes in this study drain into nearby Long Island Sound (Fig. 1A). Five of the eleven lakes are located in one of the two largest watersheds in the region; Gardner, Hayward, and Rogers drain into the Connecticut River, and Bashan and Amos drain into the Thames River. Black drains into the Quinnipiac River watershed to the west of the other study lakes. The remaining five lakes drain into smaller coastal watersheds. Quonnipaug and Bride drain into Long Island Sound through coastal streams. Pattagansett, Gorton, and Dodge are located in the same coastal watershed, where Pattagansett drains directly into Gorton, and water from Gorton and Dodge meets downstream of the two lakes before entering Long Island Sound. As a consequence of this freshwater-marine coupling among the coastal Connecticut lakes represented in the spatially explicit metacommunity analysis, freshwater hydrologic connections are likely less relevant than overland dispersal when evaluating planktonic dispersal pathways between lake pairs.

The New England coastal landscape supports lakes representing two levels of hydrologic connectivity and three food web configurations: lakes isolated from the ocean supporting food webs without alewives or with landlocked alewives, and lakes connected to the ocean where anadromous alewives are present in the food web for 6 months (Post et al. 2008). The lakes without alewives have been isolated from the ocean over recent geologic time, but are still within the same geographical region of the lakes with alewives (Fig. 1A). The lakes sampled with landlocked alewives were once connected to the ocean but became isolated by human-made dams during colonial settlement, approximately 300 years ago (Palkovacs et al. 2008). Population divergence time estimates from molecular data indicate that the landlocked alewives in these lakes were independently derived from anadromous ancestors between 5000 and 300 years before present, or 1000-60 generations of alewives (Palkovacs et al. 2008). These landlocked populations have diverged in life-history and foraging traits, including gape width and gill raker spacing, from anadromous alewives (Palkovacs and Post 2008; Post et al. 2008; Fig. 1B,C,D). Although it is unknown whether these traits are heritable, observed foraging trait differences between anadromous and landlocked alewives were maintained in a common garden experiment spanning a growing season (Palkovacs and Post 2009). Despite differences in the spatial distribution and ecological history of each lake type, there is no systematic variation in major lake characteristics (this study; one-way ANOVA, area, conductivity, depth, nitrogen, pH; lake type: *P* > 0.05, Table S1). Total phosphorus is higher in lakes with anadromous alewives relative to no alewife lakes (one-way ANOVA, lake type: *F*_2,9_ = 4.81, *P* = 0.04; Tukey's post hoc: anadromous × landlocked, *P* = 0.16; anadromous × no alewife, *P* = 0.03; landlocked × no alewife, *P* = 0.58) but not when the Massachusetts lake is excluded for the spatially explicit metacommunity analysis only encompassing the 11 Connecticut lakes (lake type: *F*_2,8_ = 3.36, *P* = 0.09; see description of the RDA analysis). Fish species richness does not differ among the three lake types (this study; one-way ANOVA, *F*_2,9_ = 0.49, *P* = 0.63). Additionally, fish community similarity in these lakes does not significantly differ by lake type (this study, excluding alewife from the analysis; PERMANOVA, Jaccard's dissimilarity, 10,000 permutations, *F*_2,11_ = 1.75, *P* = 0.10; Table S2).

### Sampling design and measurements

We sampled plankton from 12 lakes over three periods in 2009: 12 May–30 May (May), 15 June–9 July (Jun–Jul), and 10 August–27 August (Aug). Sampling periods were chosen relative to the phenology of anadromous alewife migration, where the May sampling represented lake plankton communities in the absence of anadromous alewives and the latter two sample dates represented plankton communities in the presence of anadromous alewives. Anadromous alewives begin to exert predation on zooplankton around June 15 of each year (Post et al. 2008). Assuming a 10 days generation time for large-bodied zooplankton (e.g., *Daphnia* spp.; Gillooly 2000), the latter two sampling periods encompass a minimum of eight generations and thus sufficient time to assay lake type community divergence as a function of alewife predation.

In each of the 12 lakes, we sampled crustacean zooplankton in the pelagic with an 80-*μ*m mesh plankton net and two replicate vertical tows. Tow depth was two to three meters less than the maximum lake depth. The two tow samples were pooled, and the zooplankton preserved in 70% ethanol. In the laboratory, each sample was split until there were approximately 300 cladocerans and copepods in the subsample. Zooplankton were identified to genus or species following Balcer et al. (1984) or Smith (2001) and enumerated using a dissecting microscope. Rotifers were excluded from the analysis as they are too small to be preyed upon by alewife. Ten percent of the remaining uncounted sample was subsequently scanned for new species, where individuals of each new species were enumerated. Zooplankton dry mass was estimated from measured lengths of approximately 90 individuals (median) of each species or genus and published length-mass regressions (McCauley 1984). Zooplankton were divided into two functional groups defined by body size where species with measured average maximum length ≥0.8 mm, the mean length of *Daphnia* in the study lakes, were considered large-bodied (Table S3).

We sampled phytoplankton in the pelagic with a Van Dorn bottle at three depths in the epilimnion: 0.5 m from the surface, and in the middle and lower epilimnion. The three phytoplankton samples were pooled by lake and preserved in a 0.5% glutaraldehyde solution. Subsamples of 10 or 20 mL were filtered onto mixed cellulose ester filters (Millipore, Billerica, MA) and fixed to microscope slides following Crumpton (1987). Phytoplankton were identified to genus according to Prescott (1978) or Wehr and Sheath (2003). Individual algal cells were counted at 400× magnification across a horizontal transect of 20 0.0625 mm^2^ Whipple fields using a light microscope. Cells were enumerated until a minimum of 500 individual cells, and two transects per lake were counted. Following this initial count, new genera and cells of all genera with less than 10 individuals enumerated were counted across two transects of 20 1 mm^2^ Whipple fields at 100× magnification. To account for substantial differences in cell size among taxa, biovolume was used rather than density. Biovolume was estimated according to Hillebrand et al. (1999) for each genus by lake and sampling date from up to 30 cells. Linear cell dimensions were measured from digital photographs using ImageJ (Abramoff et al. 2004). Genera were divided into two functional groups defined by size and edibility to zooplankton (Table S4). All genera with a solitary growth form and measured average maximum linear dimensions <35 *μ*m were considered edible to zooplankton (Burns 1968). In addition, the small colonial genus *Scenedesmus,* which is known to be a food source for zooplankton (e.g., Acharya et al. 2004), and similarly sized aggregate and colonial Chlorophycean genera were considered edible. All other large, colonial, and filamentous genera were considered inedible to zooplankton.

The lake environmental variables were assayed concurrently with the zooplankton and phytoplankton communities. Secchi depth was measured for each lake. Phytoplankton biomass (chlorophyll *a*), total nitrogen (TN), and total phosphorus (TP) were measured from the same depth-integrated epilimnetic water samples as those used in the phytoplankton community analysis. Phytoplankton samples were vacuum-filtered on to glass fiber filters (GF/F Whatman, Brentford, U.K.) and analyzed for chlorophyll *a* concentrations with a fluorometer (Turner Designs, Sunnyvale, CA; Marker et al. 1980). Nutrients (TN, TP) were measured after persulfate digestion on an autoanalyzer (Astoria-Pacific International, Clackamas, OR).

### Statistical analysis

We used a redundancy analysis (RDA) variation partitioning method (Borcard and Legendre 2002) to determine the amount of variation in plankton community structure explained by the local environment versus spatial processes related to species dispersal among the 11 Connecticut lakes. The total percentage of variation explained by RDA is partitioned into unique and common contributors of the environmental and spatial predictors. The abundance of zooplankton and the biovolume of phytoplankton were analyzed for each sampling period for the 11 Connecticut lakes which are located within a shared geographical region and thus represent metacommunity structure. The anadromous lake located in Massachusetts was a large spatial outlier (121 km to the nearest Connecticut lake, relative to all Connecticut lakes that are a maximum of 64 km apart from one another) and therefore excluded from the primary analysis, although a secondary analysis with all 12 lakes is reported for comparison. For the RDA results which were statistically significant in the 11 lake analysis, size-based functional groups were subsequently analyzed to identify potential subsets of species driving overall patterns. All species data were Hellinger transformed to provide unbiased estimates of variation partitioning based on RDA (Peres-Neto et al. 2006). Five environmental variables were used in each analysis and included variables likely to differentially structure plankton communities over a growing season: Secchi depth, TN, TP, and the incidence and life-history form of alewives (lake type). For zooplankton analyses, algal biomass (chl *a*) was included as the fifth predictor variable; whereas for phytoplankton analyses, zooplankton biomass was included as the fifth predictor. Environmental variables were assessed for multicollinearity prior to analysis. Lake spatial isolation was estimated from overland Euclidean distances between lake pairs, measured from geographical coordinates taken in the pelagic (ESM Table S1). Dispersal predictors were then calculated from the matrix of Euclidian distances using distance-based eigenvector maps (Griffith and Peres-Neto 2006).

Variation partitioning and spatial statistics were conducted in MATLAB 7.8 (MathWorks, Natick, MA) following procedures and code in Peres-Neto et al. (2006) and Griffith and Peres-Neto (2006). Results of variation partitioning are reported as adjusted fractions of explained variation, which in part correct for the number of environmental and spatial predictors as well as lake sample size (Peres-Neto et al. 2006). Significance of fractions was tested by 999 permutations (Borcard and Legendre 2002). For analyses where the environmental component was statistically significant (*P* ≤ 0.05), RDA in CANOCO 4.5 (ter Braak and Smilauer 2002) determined the amount of variation explained by each environmental variable while using the spatial predictors as covariables, and using 999 permutations to test for significance.

The influence of lake type, defined by the presence and life-history form of alewives, on species richness, species diversity (Shannon's index, H′), and pairwise community beta diversity (Jaccard's dissimilarity, Magurran 2004) was evaluated for zooplankton, phytoplankton, and plankton functional groups for all 12 lakes to maintain a fully replicated and balanced sampling design. Data were assessed for normality prior to repeated measures ANOVA analysis for the three sampling periods in STATISTICA 6.1 (StatSoft, Tulsa, OK). All corresponding repeated measures test results met the assumptions of sphericity. To evaluate the response of plankton community composition to lake type, a one-way permutational multivariate ANOVA was performed on zooplankton and phytoplankton for 12 lakes in PERMANOVA (Anderson 2005). Analyses were conducted on Bray–Curtis dissimilarity measures calculated from log (*x* + 1)-transformed density (zooplankton) or biovolume (phytoplankton) data for each sampling period. For statistically significant (*P* ≤ 0.05) main effects, post hoc pairwise comparisons were evaluated with the PERMANOVA *t* statistic. All tests were performed using 10,000 unrestricted permutations of the distance measure. To identify which species were responsible for significant differences in community composition by lake type based upon PERMANOVA results, similarity percentages identifying species contributing up to 90% of similarity were calculated in PRIMER 5 (Primer-E, Ivybridge, U.K.).

## Results

### Metacommunity structure

**Figure 2:**
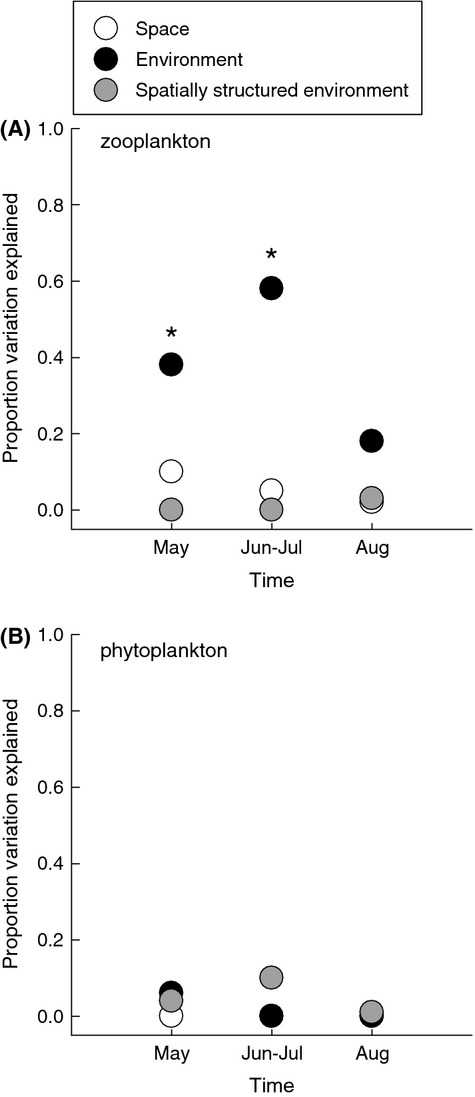
Proportion of variation explained in community composition by environmental, spatial, and spatially structured environmental predictors for (A) zooplankton and (B) phytoplankton in 11 Connecticut lakes over three sampling periods. Asterisks denote significant environmental or spatial fractions (*P* ≤ 0.05). Significance of the spatially structured environmental fraction cannot be tested.

In the 11 lake metacommunity, local environmental predictors influenced zooplankton community composition for the first two sampling periods, May (38% variation explained) and Jun–Jul (58%; Fig. 2A; Table S5). There were no significant effects of the spatial predictors on zooplankton community structure during any sampling period. Variation partitioning results from all 12 coastal lakes (including the spatial outlier in Massachusetts) were qualitatively similar in both environmental and spatial effects to the 11 lake analysis for zooplankton (Table S6). In the 11 lake metacommunity, the large-bodied zooplankton responded more strongly to environmental predictors than all zooplankton species combined during the first two sample dates, with a relative increase in the total amount of variation explained in May (environment: 64%, *P* = 0.04, space: 11%, *P* = 0.14, spatially structured environment: 0%) and Jun–Jul (environment: 67%, *P* = 0.02, space: 6%, *P* = 0.29, spatially structured environment: 0%). There was not a significant effect of the environment or space on large-bodied zooplankton in Aug (environment: 11%, *P* = 0.42, space: 5%, *P* = 0.41, spatially structured environment: 8%). Variation in the composition of small-bodied zooplankton was not influenced by the measured environmental or spatial parameters (May: environment: 17%, *P* = 0.27, space: 11%, *P* = 0.30, spatially structured environment: 0%; Jun–Jul: environment: 44%, *P* = 0.08, space: 0%, *P* = 0.46, spatially structured environment: 0%; Aug: environment: 16%, *P* = 0.29, space: 1%, *P* = 0. 47, spatially structured environment: 3%). For the phytoplankton, community structure was explained neither by the environmental variables nor by space for the 11 lake (Fig. 2B; Table S5) or 12 lake (Table S6) metacommunity.

A complementary analysis identified environmental variables important in explaining variation in community composition for all zooplankton and large-bodied zooplankton in the 11 lake metacommunity. In May, the no alewife lake type (41.1% of total variation explained by the environment, *P* = 0.006) and the landlocked alewife lake type (24.2%, *P* = 0.02) significantly contributed to the variation explained in zooplankton community composition (all other variables, *P* > 0.05). During the Jun–Jul sampling period, TP (51.4%, *P* = 0.003) explained a significant component of variation, while the anadromous lake type explained a marginally significant component (18.1%, *P* = 0.06; all other variables, *P* > 0.05). For large-bodied zooplankton, only the anadromous alewife lake type (May, 74.0%, *P* = 0.001; Jun–Jul, 70.0%, *P* = 0.002) significantly contributed to the observed variation in community structure (all other variables, *P* > 0.05).

### Diversity and composition

**Figure 3:**
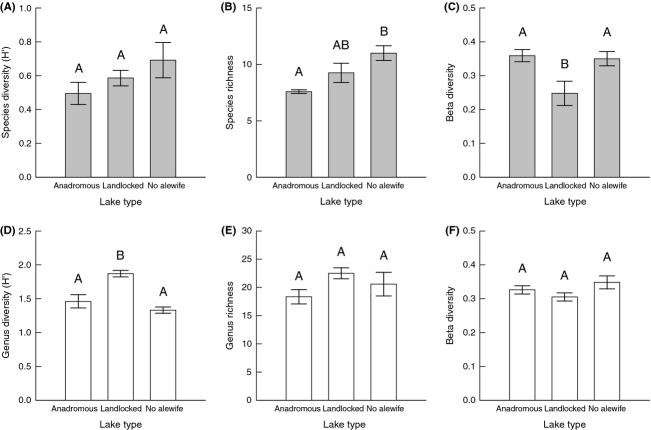
Response of zooplankton and phytoplankton (A, D) species (genus) diversity H′ (B, E), species (genus) richness, and (C, F) beta diversity (Jaccard's dissimilarity index) by lake type, defined by the presence and life-history form of alewives (*Alosa pseudoharengus*). Letters denote post hoc contrasts (*P* ≤ 0.05). Diversity values are mean ± SE of the three sampling periods; *n* = 4 lakes.

**Figure 4:**
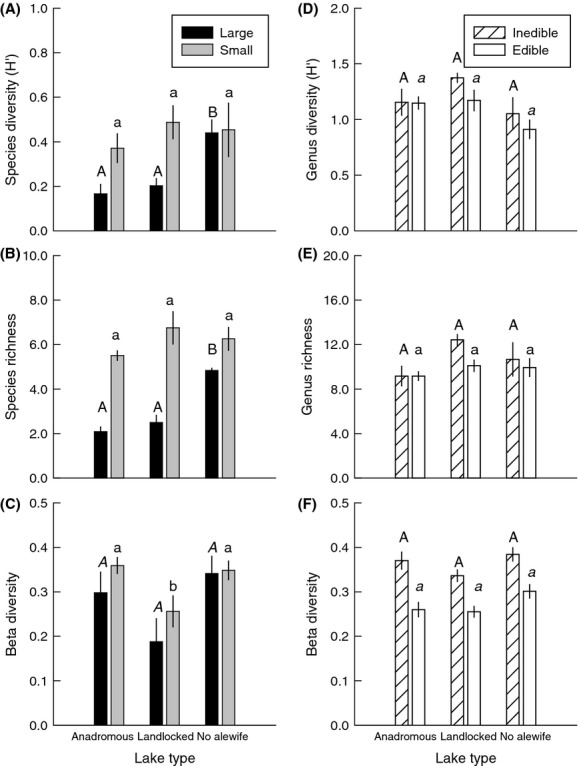
Response of zooplankton and phytoplankton functional groups (A,D) species (genus) diversity H′ (B,E), species (genus) richness, and (C, F) beta diversity (Jaccard's dissimilarity index) by lake type, defined by the presence and life-history form of alewives (*Alosa pseudoharengus*). Lowercase and upper case letters denote post hoc contrasts from separate tests by functional group (*P* ≤ 0.05). Italics indicate marginally significant contrasts *(P* ≤ 0.08). Diversity values are mean ± SE of the three sampling periods; *n* = 4 lakes.

Zooplankton species diversity (H′) did not significantly differ by lake type, defined by the presence and life-history form of alewives (Fig. 3A; Table S7). The diversity (H′) of large-bodied zooplankton, however, was greatly influenced by lake type (Fig. 4A). In the large-bodied zooplankton, species diversity was significantly higher in no alewife lakes relative to anadromous and landlocked lakes (Fig. 4A; Table S8). There was no effect of lake type on the diversity (H′) of small-bodied zooplankton.

Zooplankton species richness responded strongly to lake type (Fig. 3B; Table S7). Post hoc pairwise comparisons indicate that zooplankton communities in the absence of alewives supported the most species; whereas, communities with anadromous alewives supported the fewest species (Fig. 3B; Table S7). Zooplankton species richness in lakes with landlocked alewives did not significantly differ from lakes with anadromous alewives or without alewives. Observed patterns of local species richness were primarily driven by greater numbers of large-bodied zooplankton species in no alewife lakes (Fig. 4B; Table S8). Of the eight large-bodied zooplankton species sampled over the study period, six of the species were detected only in no alewife lakes. These six species include *Daphnia galeata mendotae*, *D. longiremis, D. pulex, Epischura lacustris*, *Leptodora kindtii*, and *Sida crystallina* (Table S3).

Zooplankton communities in landlocked lakes were more similar to one another, as illustrated by beta diversity values that were significantly lower than those in anadromous and no alewife lakes (Fig. 3C; Table S7). Small-bodied zooplankton were significant drivers of community convergence in landlocked lakes (Fig. 4C; Table S8). In contrast, patterns of beta diversity did not differ by lake type for large-bodied zooplankton. For all zooplankton diversity measures, there was no effect of sampling period in any of the analyses.

Phytoplankton genus diversity (H′) differed by lake type, where landlocked lakes supported higher levels of diversity relative to anadromous and no alewife lakes (Fig. 3D; Table S7). This response to lake type at the whole community level was not reflected in the phytoplankton functional groups defined by edibility (Fig. 4D; Table S8). Neither edible nor inedible phytoplankton diversity (H′) responded to lake type, but edible phytoplankton diversity increased through the season. Phytoplankton genus richness did not respond to lake type at the whole community level or by functional group (Figs 3E and 4E; Tables S7, S8). Beta diversity was not influenced by lake type for the entire phytoplankton community assemblage or phytoplankton functional groups (Figs 3F and 4F; Tables S7, S8). There was, however, a significant lake type by sampling period interaction affecting beta diversity in inedible phytoplankton, where landlocked and no alewife communities significantly differed from one another late in the season (Table S8).

Zooplankton community composition responded to lake type during each sampling period (Table 1). In May, prior to the presence of anadromous alewives, zooplankton community composition significantly differed between anadromous and landlocked lake types, and between landlocked and no alewife lake types. Species responsible for the majority of similarity in landlocked communities in May included the small-bodied *Bosmina longirostris* (contributing 34.37%) and the small-bodied *Diacyclops thomasi* (28.08%; Table S9). In anadromous lakes in May, the species that contributed most were the large-bodied *Daphnia ambigua* (46.10%) and the small-bodied *Bosmina longirostris* (23.67%). The species that contributed most to similarity in no alewife lakes in May was the large-bodied *Skistodiaptomus* (62.06%), with each remaining species contributing less than 10%. During Jun–Jul, in the presence of anadromous alewives, zooplankton community composition between anadromous and no alewife lakes significantly differed, and there was a marginal difference between landlocked and no alewife lakes (*P* = 0.056; Table 1). In landlocked lakes, the small-bodied *Bosmina longirostris* (39.91%) again contributed the most to similarity, with the large-bodied *Skistodiaptomus* (19.78%) being the second greatest contributor (Table S9). Anadromous lakes in Jun–Jul notably differed from May, where *Bosmina longirostris* (79.92%) primarily contributed to the dominant patterns of similarity (rather than *Daphnia ambigua*), with each remaining species contributing less than 10%. As in May, *Skistodiaptomus* (47.99%) contributed most to similarity along with *Diaphanosoma* (20.14%) in no alewife lakes.

**Table 1:** Effects of lake type, defined by the presence and life-history form of alewives (*Alosa pseudoharengus*), on zooplankton and phytoplankton community composition for each of three sampling periods, as analyzed with PERMANOVA.

Response variable	df	*F*	*P*-value	Contrasts
Zooplankton				
May				
Lake type	2, 9	3.53	*0.005*	(A, L*), (L, N*)
Jun–Jul				
Lake type	2, 9	2.81	*0.02*	(A, N*), (L, N†)
Aug				
Lake type	2, 9	1.84	*0.04*	(A, N*)
Phytoplankton				
May				
Lake type	2, 9	1.49	0.12	
Jun–Jul				
Lake type	2, 9	1.11	0.35	
Aug				
Lake type	2, 9	1.30	*0.04*	(A, L†), (A, N*)

Abbreviations for post hoc contrasts: A, anadromous alewife; L, landlocked alewife; N, no alewife.

Significant probability values in italics. Post hoc pairwise comparisons (PERMANOVA *t*) are reported for significant main effects.

Contrasts significance levels: †*P* = 0.056, *P < 0.05.

In Aug, only anadromous and no alewife lakes supported zooplankton communities that significantly differed in composition (Table 1). In anadromous lakes, *Bosmina longirostris* (68.29%) and *Eubosmina* (14.56%) explained the majority of similarity (Table S9). The species that contributed most to homogeneity within the no alewife lakes in Aug were the copepods *Skistodiaptomus* (39.63%) and *Mesocyclops edax* (24.31%).

Phytoplankton community composition differed by lake type only in the Aug sampling period (Table 1). Phytoplankton communities in anadromous lakes significantly differed from those in lakes without alewives. Additionally, community composition differed between anadromous and landlocked lake types (*P* = 0.056). In anadromous lakes, phytoplankton genera that contributed most to similarity were the edible *Gymnodinium* (contributing 28.17%) and edible *Cyclotella* (24.26%; Table S10). The genera that contributed most to similarity in landlocked lakes were the inedible *Peridinium* (21.76%) and inedible *Ceratium* (15.62%). The edible *Cryptomonas* (29.67%) and *Ceratium* (17.99%) contributed most to similarity in no alewife lakes.

## Discussion

Intraspecific phenotypic variation in a predator strongly structured multitrophic diversity and composition in the lake plankton metacommunity. The incidence and magnitude of the response, however, varied by trophic level and planktonic functional group. The local zooplankton communities were structured by species sorting from predation, and to a lesser extent phosphorus limitation. Differential residency and selective predation by anadromous and landlocked alewives maintained a spatio-temporal mosaic of patch occupancy by large-bodied taxa. Intraspecific variation in alewives altered local zooplankton species richness and beta diversity, with landlocked alewife lake communities exhibiting intermediate richness between species-poor anadromous and species-rich no alewife lakes, and greater community similarity than within either anadromous alewife or no alewife lakes. Phytoplankton diversity also responded to intraspecific variation in alewives, where genus diversity was highest in landlocked alewife lakes. The phytoplankton communities responded to intraspecific variation late in the growing season, when contrasting indirect predator effects influence the observed impacts on local composition. Taken together, the results suggest that zooplankton and phytoplankton dispersal distances were large enough for species to arrive to each lake in the region, where species subsequently sorted in response to intraspecific variation in alewives. This local environmental structuring of plankton in the absence of any spatial influence on community composition suggests species sorting operates in the metacommunity. The results are among the first to highlight an important role for intraspecific variation in altering local and beta diversity in multitrophic metacommunities.

Species sorting structured lake zooplankton communities, where intraspecific variation in alewife lake residency and foraging trait morphology influenced local composition in the metacommunity. Zooplankton dispersal in the region was neither limiting nor high at the spatial scale encompassed in the study and delivered a migrant species pool suited to the local environment of each lake type, yielding consistent community composition relative to lake food web structure (Cottenie and De Meester 2004; Howeth and Leibold 2008). Other observational studies comparing roles of environmental and spatial processes in structuring zooplankton communities similarly find the local environment to be relatively more important in affecting composition (Beisner et al. 2006; Strecker et al. 2008). Zooplankton community composition was most clearly differentiated by lake type in the spring, prior to anadromous alewife predation, when *Daphnia* are abundant only in no alewife and anadromous lakes (Fig. 2, Table 1). Community composition began to converge in late summer, when intense young-of-year planktivory from all fish species reduces *Daphnia* densities in the three lake types (Post et al. 2008), and phosphorus becomes a limiting nutrient. The strong patterns of community differentiation by lake type in spring to mid-summer were primarily driven by large-bodied zooplankton, the preferred prey of alewives (Brooks and Dodson 1965; Palkovacs and Post 2008). In these coastal lakes, differential predation by anadromous and landlocked alewives on large-bodied zooplankton maintains a spatio-temporal mosaic of patch occupancy by large-bodied taxa as a function of food web architecture.

The predation gradient across the three different food web configurations in the coastal lake metacommunity results in alewife-dependent sorting maintaining distinct patterns of zooplankton diversity in the metacommunity. Local zooplankton species richness was greatest in the absence of alewives and reduced in the presence of the strongly size-selective anadromous alewives (Fig. 3). This result in natural lake ecosystems reflects previous differences in zooplankton species richness observed among the three lake types in experimental mesocosms (Palkovacs and Post 2009). In this study, six large-bodied species, including three *Daphnia* spp., were maintained exclusively in no alewife lakes. The no alewife lakes thus serve as a spatial refuge and are responsible for maintaining the majority of zooplankton species and alewife prey diversity in the regional species pool. Landlocked lakes had low beta diversity and strong community convergence driven by small-bodied species, primarily *Bosmina longirostris* (Fig. 4C, Table S9). The constant environment created by landlocked alewife predation likely prevented local species turnover and changes in planktonic community composition. The derived landlocked life-history form of alewife thus differentially affects spatial community turnover and increases community homogeneity in the landscape relative to the two ancestral lake types, no alewife, and anadromous alewife lakes. The influence of landlocked alewife on beta diversity was likely independent of any effects on alpha diversity, as local species richness in landlocked lakes was intermediate to the other two lake types (as opposed to highest, as it would be if alpha diversity affected beta diversity following the prediction of Chase et al. 2011). The effect of predation in maintaining patch-type heterogeneity in the landscape has been demonstrated previously but with single, functionally homogeneous species including invertebrate (Kneitel and Miller 2003) and vertebrate top predators (Howeth and Leibold 2010b, 2013) in experimental metacommunities, and with fish predators in natural pond metacommunities (Chase et al. 2009). These studies showed species sorting and reduced species diversity in the presence of predators over a broad range of dispersal rates. Our findings build upon this work and suggest that phenotypic diversification *within* predator species can also be ecologically important and can strongly structure local communities within a shared geographical region composed of moderately connected communities.

Differences in lake residency and prey selectivity of the two alewife life-history forms yield a mosaic of contrasting indirect predator effects influencing phytoplankton diversity and composition in the landscape. The high phytoplankton genus diversity observed in lakes with landlocked alewives likely results from press alewife predation catalyzing constant grazing release from large-bodied daphniids and copepods. Neither the combined environmental variables nor dispersal limitation or mass effects influenced phytoplankton community structure. The result is in contrast to strong environmental sorting observed using the same variation partitioning approach in diatom metacommunities encompassing multiple geographical regions and spanning a range of spatial scales (Verleyen et al. 2009), and in highly interconnected ponds where *Daphnia* and macrophyte cover affected phytoplankton composition (Vanormelingen et al. 2008). Species sorting in phytoplankton in this study, however, is apparent in the response of composition to alewives. The indirect predator effects by alewife lake type on phytoplankton community composition are in part temporally dependent and strongest late in the growing season. The effects of the two alewife life-history forms on phytoplankton composition align with previously documented differences in the strength of trophic cascades caused by the two different alewife forms (Post et al. 2008; Weis and Post 2013). In anadromous lakes, where trophic cascades are strongest (Post et al. 2008), edible phytoplankton (*Cyclotella, Gymnodinium*) dominated communities; whereas in landlocked lakes, inedible genera (*Ceratium, Peridinium*) shaped community structure. The rarity of *Daphnia* in anadromous lakes in summer facilitates grazing release and an increase in the biomass of edible phytoplankton (Post et al. 2008). The contrasting influence of two alewife life-history forms on zooplankton and phytoplankton community structure suggests that intraspecific variation in a predator can influence diversity and composition of multiple trophic levels when dispersal allows species to track direct and indirect predator effects, but is sufficiently low to prevent mass effects.

The finding of species sorting and low rates of plankton dispersal in the region assists interpretation of divergent ecological and evolutionary patterns by alewife lake type previously observed in the metacommunity. Species sorting in zooplankton facilitates compositional tracking of functional differences in the two life-history forms of alewife and likely promotes the hypothesized eco-evolutionary feedbacks occurring between landlocked alewife foraging traits and zooplankton community structure (Palkovacs and Post 2009; Post and Palkovacs 2009). Eco-evolutionary feedbacks may occur when constant size-selective predation yields a small-bodied zooplankton community which then feeds back to select for narrower gill raker spacing in landlocked alewives (Fig. 1C and D). If dispersal rates were high in the region, mass effects could prevent alewife from structuring the prey community by facilitating colonization of large-bodied species (e.g., *Daphnia*) continuously maintained in the no alewife lakes. In this scenario, source-sink dynamics would maintain large-bodied prey in lakes with landlocked alewives and prevent eco-evolutionary feedbacks from developing. The species sorting observed in zooplankton may also have consequences for adaptive sorting in the metacommunity (Urban et al. 2008). Documented differential evolution in *Daphnia ambigua* populations in lakes with anadromous and landlocked alewives could only occur under migration rates low enough to prevent swamping of evolutionary processes (Walsh and Post 2011). Such eco-evolutionary consequences of prey dispersal rates in the region could be investigated in similar ecosystems under varying levels of connectivity. For example, in the Great Lakes, where dispersal rates of landlocked alewives and zooplankton are higher from hydrologic connectivity (Wells 1970), there may be no opportunity for eco-evolutionary feedbacks. Ultimately, metacommunity dynamics of prey may moderate ecological and evolutionary potential within predator populations and reciprocal selection on prey communities, in part influencing hot and cold spots of species and ecological diversity across biogeographical regions (e.g., Benkman 1999; Thompson 2005).

Two methodological limitations should be taken into account when interpreting our statistical variation partitioning results in the context of metacommunity structure. First, we did not measure or include all possible environmental variables that may be important in affecting zooplankton and phytoplankton community composition. For example, previous studies have found dissolved inorganic carbon, dissolved organic carbon, and pH to be important environmental drivers of zooplankton community structure in temperate lake ecosystems (Beisner et al. 2006; Strecker et al. 2008; Derry et al. 2009). This limitation makes our interpretation of the relative importance of local environmental conditions in determining zooplankton community structure conservative and would further reduce the importance of a spatial component. Second, the role of dispersal in structuring communities was inferred indirectly via the significance of the spatial component in the variation partitioning analysis. Direct measures of different modes of passive planktonic dispersal (i.e., aerial, anthropogenic, water) prove logistically challenging across multiple lake ecosystems and were beyond the scope of this study. Other indirect measures of planktonic dispersal, however, such as estimates of gene flow and population genetic differentiation among lakes using molecular markers would be useful in elucidating the magnitude of species dispersal among lakes (Derry et al. 2009; Pantel et al. 2011a). Future work addressing comparative landscape genetics of multiple zooplankton and phytoplankton species in the region could provide additional insight into dispersal and any possible influence of historical biogeography on multitrophic metacommunity structure.

This is one of the first studies to acknowledge the influence of intraspecific phenotypic variation in a predator on multitrophic metacommunity structure. The results suggest that low rates of zooplankton dispersal in the region supply a migrant pool that is structured by local environmental processes within lakes, with intraspecific variation in alewives being the most important variable of those evaluated. Species sorting in zooplankton in response to the two life-history forms of alewives is apparent in local and beta diversity, and consistent community composition by lake type. Sorting in the phytoplankton is reflected by the differential diversity response to anadromous and landlocked alewives, and composition late in the growing season. Collectively, the results indicate that intraspecific variation in a key predator maintains a spatial mosaic of contrasting patterns of multitrophic diversity and composition in a moderately connected metacommunity. Recent work with model vertebrate predators such as Trinidadian guppies (*Poecilia reticulata*; Bassar et al. 2010) and three-spine sticklebacks (*Gasterosteus aculeatus*; Harmon et al. 2009) also finds that intraspecific variation can influence community structure. This study expands upon these concepts focused at the local scale and suggests that integrating patterns and mechanisms from local to regional spatial scales can be important to better interpret underlying ecological and evolutionary processes occurring among multiple trophic levels in response to intraspecific diversification in predators.

## Supporting Information

**Table S1.** Basic lake environmental variables for the 12 study lakes classified by the presence and life-history form of alewives (*Alosa pseudoharengus*): anadromous alewife, landlocked alewife, or no alewife.

**Table S2**. Fish community composition for 12 study lakes classified by the presence and life-history form of alewives (*Alosa pseudoharengus*): anadromous alewife, landlocked alewife, or no alewife.

**Table S3.** Zooplankton species in the study, with body size classification (large, mean length ≥0.8 mm; small <0.8 mm) and average densities per liter (1 SE) by lake type, defined by the presence and life-history form of alewives (*Alosa pseudoharengus*), from three sampling periods ranging from 12 May 2009 to 27 August 2009.

**Table S4.** Phytoplankton genera in the study, with growth form and edibility of the genus to zooplankton (edible, ≤35 μm; inedible, >35 μm) noted.

**Table S5.** Variation partitioning of community composition using both environmental [E] and spatial [S] predictors for zooplankton and phytoplankton metacommunity structure in 11 Connecticut lakes.

**Table S6.** Variation partitioning of community composition using both environmental [E] and spatial [S] predictors for zooplankton and phytoplankton metacommunity structure in 12 coastal lakes (11 Connecticut lakes, same Connecticut lakes as reported in Table S5, in addition to 1 Massachusetts lake).

**Table S7.** Effects of lake type, defined by the presence and life-history form of alewives (*Alosa pseudoharengus*), on species diversity, species richness, and beta diversity of zooplankton and phytoplankton, as analyzed with repeated measures ANOVA.

**Table S8.** Effects of lake type, defined by the presence and life-history form of alewives (*Alosa pseudoharengus*), on species diversity, species richness, and beta diversity of zooplankton and phytoplankton functional groups, as analyzed with repeated measures ANOVA.

**Table S9.** Species contributing to the similarity of zooplankton communities by lake type, defined by the presence and life-history form of alewives (*Alosa pseudoharengus*), during the May, June–July, and August 2009 sampling periods.

**Table S10.** Genera contributing to the similarity of phytoplankton communities by lake type, defined by the presence and life-history form of alewives (*Alosa pseudoharengus*), during the August 2009 sampling period.
