# Supplementary material for: Intraspecific phenotypic variation in a fish predator affects multitrophic lake metacommunity structure
Source: Ecol Evol. 2013 Nov 14;3(15):5031–44. doi: 10.1002/ece3.878 (PMC3892366; doi:10.1002/ece3.878)
Supplement: Supplementary file 1 [file ece30003-5031-SD1.doc]

**Supporting Information**

Intraspecific phenotypic variation in a fish predator affects multi-trophic lake metacommunity structure

Jennifer G. Howeth^1,2,4^ , Jerome J. Weis^1^, Jakob Brodersen^1,3^, Elizabeth C. Hatton^1^, David M. Post^1^

^1^ Department of Ecology and Evolutionary Biology, Yale University, New Haven, Connecticut 06520-8105, USA

^2^ Current address: Department of Biological Sciences, University of Alabama, Tuscaloosa, Alabama 35487-0206, USA

^3^ Current address: Department of Fish Ecology and Evolution, EAWAG Swiss Federal Institute of Aquatic Science and Technology, Center of Ecology, Evolution and Biochemistry, Seestrasse 79, CH-6047 Kastanienbaum, Switzerland

^4^ Corresponding author, e-mail: jghoweth@as.ua.edu; phone: 205-348-0865

**Table S1.** Basic lake environmental variables for the 12 study lakes classified by the presence and life-history form of alewives (*Alosa pseudoharengus*): anadromous alewife, landlocked alewife, or no alewife. Conductivity and pH values were sampled once for each lake between 10 August 2009 and 27 August 2009. Total nitrogen (TN) and total phosphorus (TP) represent the average values over the three sample dates for each lake, ranging from 12 May 2009 to 27 August 2009. Spatial lake position is represented by the geographic coordinates of the pelagic habitat.

|  |  |  |  |  |  |  |  |  |  |
| --- | --- | --- | --- | --- | --- | --- | --- | --- | --- |
| Lake | Lake type | Latitude  (DD) | Longitude  (DD) | Area  (ha) | Conductivity  (μS) | Max depth  (m) | pH | TN  (mg/L) | TP  (μg/L) |
|  |  |  |  |  |  |  |  |  |  |
|  |  |  |  |  |  |  |  |  |  |
| Amos | Landlocked | 41.516 | -71.975 | 42.0 | 124.2 | 13.7 | 7.93 | 0.44 | 23.60 |
|  |  |  |  |  |  |  |  |  |  |
| Bashan | No alewife | 41.491 | -72.411 | 110.5 | 72.7 | 14.3 | 6.82 | 0.22 | 7.83 |
|  |  |  |  |  |  |  |  |  |  |
| Black | No alewife | 41.524 | -72.743 | 30.2 | 202.7 | 7.0 | 8.05 | 0.37 | 18.82 |
|  |  |  |  |  |  |  |  |  |  |
| Bride | Anadromous | 41.327 | -72.237 | 28.7 | 115.0 | 10.7 | 7.85 | 0.43 | 23.98 |
|  |  |  |  |  |  |  |  |  |  |
| Dodge | Anadromous | 41.328 | -72.198 | 13.9 | 98.9 | 15.5 | 7.26 | 0.50 | 18.77 |
|  |  |  |  |  |  |  |  |  |  |
| Gardner | No alewife | 41.523 | -72.222 | 194.7 | 85.1 | 13.4 | 7.39 | 0.40 | 18.33 |
|  |  |  |  |  |  |  |  |  |  |
| Gorton | Anadromous | 41.339 | -72.209 | 21.5 | 106.7 | 3.4 | 8.16 | 0.61 | 27.43 |
|  |  |  |  |  |  |  |  |  |  |
| Great Herring‡ | Anadromous | 41.796 | -70.563 | 152.2 | 89.5 | 12.8 | 8.24 | 0.38 | 24.68 |
|  |  |  |  |  |  |  |  |  |  |
| Hayward | No alewife | 41.518 | -72.327 | 79.6 | 95.2 | 10.7 | 7.57 | 0.36 | 11.87 |
|  |  |  |  |  |  |  |  |  |  |
| Pattagansett | Landlocked | 41.373 | -72.230 | 49.2 | 75.1 | 10.1 | 7.27 | 0.36 | 15.28 |
|  |  |  |  |  |  |  |  |  |  |
| Quonnipaug | Landlocked | 41.390 | -72.687 | 44.6 | 160.6 | 14.5 | 8.59 | 0.28 | 15.77 |
|  |  |  |  |  |  |  |  |  |  |
| Rogers | Landlocked | 41.364 | -72.230 | 106.0 | 56.1 | 20.0 | 7.45 | 0.33 | 15.07 |
|  |  |  |  |  |  |  |  |  |  |

**Table S1.**

‡ TN and TP for the first sample period represented by 10 May 2010 data

**Table S2**. Fish community composition for 12 study lakes classified by the presence and life-history form of alewives (*Alosa pseudoharengus*): anadromous alewife, landlocked alewife, or no alewife. Fish species were considered present in the lake if sampled at least once over three sample dates ranging from 12 May 2009 to 27 August 2009. Fish were sampled on the same dates as the plankton and environmental variables. Littoral and pelagic habitats were sampled concurrently for fish, where electrofishing was employed in the littoral, and gill nets and purse seines were employed in the pelagic. Detailed methods can be found in Brodersen, Howeth, and Post, *in preparation*.

**Table 1.**

|  |  | | | |  | | | |  | | | |
| --- | --- | --- | --- | --- | --- | --- | --- | --- | --- | --- | --- | --- |
|  | Anadromous | | | | Landlocked | | | | No alewife | | | |
|  |  | | | |  | | | |  | | | |
|  |  |  |  |  |  |  |  |  |  |  |  |  |
|  | Bride | Dodge | Gorton | Great Herring | Amos | Pattagansett | Quonnipaug | Rogers | Bashan | Black | Gardner | Hayward |
|  |  |  |  |  |  |  |  |  |  |  |  |  |
|  |  |  |  |  |  |  |  |  |  |  |  |  |
| *Alosa pseudoharengus* | x | x | x | x | x | x | x | x |  |  |  |  |
|  |  |  |  |  |  |  |  |  |  |  |  |  |
| *Ameiurus natalis* |  |  |  |  |  |  |  | x |  | x |  |  |
|  |  |  |  |  |  |  |  |  |  |  |  |  |
| *Ameiurus nebulosus* |  |  | x | x | x |  | x | x | x | x | x |  |
|  |  |  |  |  |  |  |  |  |  |  |  |  |
| *Catostomus commersoni* | x |  |  | x |  |  |  |  |  |  | x |  |
|  |  |  |  |  |  |  |  |  |  |  |  |  |
| *Esox niger* | x | x | x |  | x | x | x | x | x | x | x | x |
|  |  |  |  |  |  |  |  |  |  |  |  |  |
| *Fundulus diaphanus* | x |  |  | x |  |  |  |  | x |  |  |  |
|  |  |  |  |  |  |  |  |  |  |  |  |  |
| *Lepomis auritus* |  |  |  |  |  |  | x |  | x |  |  |  |
|  |  |  |  |  |  |  |  |  |  |  |  |  |
| *Lepomis gibbosus* | x | x | x | x | x | x | x | x | x | x | x | x |
|  |  |  |  |  |  |  |  |  |  |  |  |  |
| *Lepomis macrochirus* | x | x | x | x | x | x | x | x | x | x | x | x |
|  |  |  |  |  |  |  |  |  |  |  |  |  |
| *Micropterus dolomieu* |  |  |  | x |  |  |  |  | x |  | x | x |
|  |  |  |  |  |  |  |  |  |  |  |  |  |
| *Micropterus salmoides* | x | x | x |  | x | x | x | x | x | x | x | x |
|  |  |  |  |  |  |  |  |  |  |  |  |  |
| *Morone americana* | x | x |  | x |  |  |  | x |  |  |  |  |
|  |  |  |  |  |  |  |  |  |  |  |  |  |
| *Notemigonus crysoleucus* |  |  | x |  | x | x | x | x |  | x | x | x |
|  |  |  |  |  |  |  |  |  |  |  |  |  |
| *Notropis bifrenatus* | x |  | x |  |  |  | x | x | x | x |  | x |
|  |  |  |  |  |  |  |  |  |  |  |  |  |
| *Perca flavescens* | x | x | x | x | x | x | x | x | x | x | x | x |
|  |  |  |  |  |  |  |  |  |  |  |  |  |
| *Pomoxis nigromaculatus* |  | x | x |  |  | x | x | x | x | x | x | x |
|  |  |  |  |  |  |  |  |  |  |  |  |  |
| *Umbra limi* |  |  |  |  |  |  |  |  |  | x |  |  |
|  |  |  |  |  |  |  |  |  |  |  |  |  |

**Table S2.**

**Table S3.** Zooplankton species in the study, with body size classification (large, mean length ≥ 0.8 mm; small < 0.8 mm) and average densities per litre (1 SE) by lake type, defined by the presence and life-history form of alewives (*Alosa pseudoharengus*), from three sampling periods ranging from 12 May 2009 to 27 August 2009.

**Table S3.**

|  |  |  |  |  |  |
| --- | --- | --- | --- | --- | --- |
| Species | Order | Size | Anadromous | Landlocked | No alewife |
|  |  |  |  |  |  |
|  |  |  |  |  |  |
| *Acanthocyclops vernalis* | Cyclopoida | Small | 2.13 (2.05) | 1.83 (1.35) | 0.41 (0.50) |
|  |  |  |  |  |  |
| *Alona* | Cladocera | Small | 0.008 (0.009) | 0.002 (0.004) | 0.007 (0.008) |
|  |  |  |  |  |  |
| *Bosmina longirostris* | Cladocera | Small | 10.86 (6.43) | 6.62 (3.34) | 1.40 (1.11) |
|  |  |  |  |  |  |
| *Camptocercus* | Cladocera | Small | 0 | 0 | 0.001 (0.001) |
|  |  |  |  |  |  |
| *Ceriodaphnia lacustris* | Cladocera | Small | 0.12 (0.14) | 1.56 (1.12) | 0.63 (0.58) |
|  |  |  |  |  |  |
| *Chydorus sphaericus* | Cladocera | Small | 0.02 (0.03) | 0.45 (0.54) | 2.38 (2.60) |
|  |  |  |  |  |  |
| *Daphnia ambigua* | Cladocera | Large | 4.26 (3.66) | 0.02 (0.03) | 0.64 (0.64) |
|  |  |  |  |  |  |
| *Daphnia catawba* | Cladocera | Large | 0.24 (0.43) | 0 | 0.53 (0.33) |
|  |  |  |  |  |  |
| *Daphnia galeata mendotae* | Cladocera | Large | 0 | 0 | 1.09 (1.58) |
|  |  |  |  |  |  |
| *Daphnia longiremis* | Cladocera | Large | 0 | 0 | 0.16 (0.14) |
|  |  |  |  |  |  |
| *Daphnia pulex* | Cladocera | Large | 0 | 0 | 0.32 (0.26) |
|  |  |  |  |  |  |
| *Diacyclops thomasi* | Cyclopoida | Small | 0.38 (0.32) | 2.01 (2.06) | 0.82 (0.85) |
|  |  |  |  |  |  |
| *Diaphanosoma* | Cladocera | Small | 0.58 (0.52) | 0.31 (0.18) | 1.71 (1.31) |
|  |  |  |  |  |  |
| *Epischura lacustris* | Calanoida | Large | 0 | 0 | 0.10 (0.10) |
|  |  |  |  |  |  |
| *Eubosmina* | Cladocera | Small | 1.50 (0.85) | 0.02 (0.03) | 1.48 (1.44) |
|  |  |  |  |  |  |
| *Eurycercus* | Cladocera | Small | 0 | 0.005 (0.006) | 0 |
|  |  |  |  |  |  |
| *Holopedium gibberum* | Cladocera | Small | 0.02 (0.03) | 0.14 (0.12) | 0.03 (0.03) |
|  |  |  |  |  |  |
| *Leptodora kindtii* | Cladocera | Large | 0 | 0 | 0.01 (0.02) |
|  |  |  |  |  |  |
| *Mesocyclops edax* | Cyclopoida | Large | 0.49 (0.33) | 0.48 (0.31) | 1.44 (0.62) |
|  |  |  |  |  |  |
| *Sida crystallina* | Cladocera | Large | 0 | 0 | 0.002 (0.003) |
|  |  |  |  |  |  |
| *Skistodiaptomus* | Calanoida | Large | 0.43 (0.48) | 0.95 (0.53) | 4.39 (1.34) |
|  |  |  |  |  |  |
| *Tropocyclops extensus* | Cyclopoida | Small | 0.28 (0.46) | 0.15 (0.08) | 0.27 (0.13) |
|  |  |  |  |  |  |

**Table S4.** Phytoplankton genera in the study, with growth form and edibility of the genus to zooplankton (edible, ≤35μm; inedible, >35 μm) noted.

|  |  |  |  |
| --- | --- | --- | --- |
| Genus | Phylum | Growth Form | Edibility |
|  |  |  |  |
|  |  |  |  |
| *Achnanthes* | Bacillariophyta | Solitary | Edible |
|  |  |  |  |
| *Anabaena* | Nostocales | Filamentous | Inedible |
|  |  |  |  |
| *Ankistrodesmus* | Chlorophyta | Solitary | Edible |
|  |  |  |  |
| *Aphanocapsa* | Chroococcales | Colonial | Inedible |
|  |  |  |  |
| *Asterionella* | Bacillariophyta | Colonial | Inedible |
|  |  |  |  |
| *Botryococcus* | Chlorophyta | Colonial | Inedible |
|  |  |  |  |
| *Ceratium* | Dinophyta | Solitary | Inedible |
|  |  |  |  |
| *Chlamydomonas* | Chlorophyta | Solitary | Edible |
|  |  |  |  |
| *Chlorella* | Chlorophyta | Aggregate | Edible |
|  |  |  |  |
| *Chroococcus* | Chroococcales | Aggregate | Inedible |
|  |  |  |  |
| *Closterium* | Chlorophyta | Solitary | Inedible |
|  |  |  |  |
| *Cocconeis* | Bacillariophyta | Solitary | Inedible |
|  |  |  |  |
| *Cosmarium* | Chlorophyta | Solitary | Edible |
|  |  |  |  |
| *Crucigenia* | Chlorophyta | Colonial | Edible |
|  |  |  |  |
| *Cryptomonas* | Cryptophyta | Solitary | Edible |
|  |  |  |  |
| *Cyclotella* | Bacillariophyta | Solitary | Edible |
|  |  |  |  |
| *Cymbella* | Bacillariophyta | Solitary | Inedible |
|  |  |  |  |
| *Dictyosphaerium* | Chlorophyta | Colonial | Inedible |
|  |  |  |  |
| *Dinobryon* | Chrysophyta | Colonial | Inedible |
|  |  |  |  |
| *Eudorina* | Chlorophyta | Colonial | Edible |
|  |  |  |  |
| *Euglena* | Euglenophyta | Solitary | Inedible |
|  |  |  |  |
| *Fragilaria* | Bacillariophyta | Colonial | Inedible |
|  |  |  |  |
| *Frustulia* | Bacillariophyta | Solitary | Inedible |
|  |  |  |  |
| *Gomphonema* | Bacillariophyta | Solitary | Edible |
|  |  |  |  |
| *Gymnodinium* | Dinophyta | Solitary | Edible |
|  |  |  |  |
| *Gyrosigma* | Bacillariophyta | Solitary | Inedible |
|  |  |  |  |
| *Melosira* | Bacillariophyta | Filamentous | Inedible |
|  |  |  |  |
| *Merismopedia* | Chroococcales | Colonial | Inedible |
|  |  |  |  |
| *Microcystis* | Chroococcales | Colonial | Inedible |
|  |  |  |  |
| *Mougeotia* | Chlorophyta | Filamentous | Inedible |
|  |  |  |  |
| *Navicula* | Bacillariophyta | Solitary | Edible |
|  |  |  |  |
| *Oocystis* | Chlorophyta | Colonial | Edible |
|  |  |  |  |
| *Oscillatoria* | Oscillatoriales | Filamentous | Inedible |
|  |  |  |  |
| *Palmella* | Chlorophyta | Colonial | Inedible |
|  |  |  |  |
| *Pediastrum* | Chlorophyta | Colonial | Edible |
|  |  |  |  |
| *Peridinium* | Dinophyta | Solitary | Inedible |
|  |  |  |  |
| *Pinnularia* | Bacillariophyta | Solitary | Inedible |
|  |  |  |  |
| *Poterioochromonas* | Chrysophyta | Aggregate | Inedible |
|  |  |  |  |
| *Protoderma* | Chlorophyta | Colonial | Edible |
|  |  |  |  |
| *Scenedesmus* | Chlorophyta | Colonial | Edible |
|  |  |  |  |
| *Selenastrum* | Chlorophyta | Solitary | Edible |
|  |  |  |  |
| *Spirulina* | Oscillatoriales | Filamentous | Inedible |
|  |  |  |  |
| *Spondylosium* | Chlorophyta | Filamentous | Inedible |
|  |  |  |  |
| *Staurastrum* | Chlorophyta | Solitary | Inedible |
|  |  |  |  |
| *Staurodesmus* | Chlorophyta | Solitary | Edible |
|  |  |  |  |
| *Synedra* | Bacillariophyta | Solitary | Inedible |
|  |  |  |  |
| *Tabellaria* | Bacillariophyta | Colonial | Inedible |
|  |  |  |  |
| *Tetraedron* | Chlorophyta | Solitary | Edible |
|  |  |  |  |
| *Trachelomonas* | Euglenophyta | Solitary | Edible |
|  |  |  |  |
| *Uroglena* | Chrysophyta | Colonial | Inedible |
|  |  |  |  |
| *Volvox* | Chlorophyta | Colonial | Inedible |
|  |  |  |  |
| *Woronichinia* | Chroococcales | Colonial | Inedible |
|  |  |  |  |

**Table S5.** Variation partitioning of community composition using both environmental [E] and spatial [S] predictors for zooplankton and phytoplankton metacommunity structure in 11 Connecticut lakes.

|  |  |  |  |  |  |
| --- | --- | --- | --- | --- | --- |
| Sample | [E‌│S]  Adj. *R*^2^ | [E‌│S]  *P* | [S‌│E]  Adj. *R*^2^ | [S‌│E]  *P* | [E‌+S]  Adj. *R*^2^ |
|  |  |  |  |  |  |
|  |  |  |  |  |  |
| Zooplankton |  |  |  |  |  |
|  |  |  |  |  |  |
| May | 0.38 | *0.05* | 0.10 | 0.19 | 0 |
|  |  |  |  |  |  |
| Jun-Jul | 0.58 | *0.02* | 0.05 | 0.32 | 0 |
|  |  |  |  |  |  |
| Aug | 0.18 | 0.27 | 0.02 | 0.44 | 0.03 |
|  |  |  |  |  |  |
| Phytoplankton |  |  |  |  |  |
|  |  |  |  |  |  |
| May | 0.06 | 0.44 | 0 | 0.53 | 0.04 |
|  |  |  |  |  |  |
| Jun-Jul | 0 | 0.69 | 0 | 0.67 | 0.10 |
|  |  |  |  |  |  |
| Aug | 0 | 0.56 | 0 | 0.74 | 0.01 |
|  |  |  |  |  |  |

[E‌│S], variation explained by pure environmental variables, contribution of S removed.

[S‌│E], variation explained by pure spatial variables, contribution of E removed.

[E‌+S], variation explained by both environment and space (spatially structured environment). Significance of this fraction cannot be tested.

Significant probability values in italics.

**Table S6.** Variation partitioning of community composition using both environmental [E] and spatial [S] predictors for zooplankton and phytoplankton metacommunity structure in 12 coastal lakes (11 Connecticut lakes, same Connecticut lakes as reported in Table S5, in addition to 1 Massachusetts lake).

|  |  |  |  |  |  |
| --- | --- | --- | --- | --- | --- |
| Sample | [E‌│S]  Adj. *R*^2^ | [E‌│S]  *P* | [S‌│E]  Adj. *R*^2^ | [S‌│E]  *P* | [E‌+S]  Adj. *R*^2^ |
|  |  |  |  |  |  |
|  |  |  |  |  |  |
| Zooplankton |  |  |  |  |  |
|  |  |  |  |  |  |
| May | 0.34 | *0.03* | 0.03 | 0.29 | 0 |
|  |  |  |  |  |  |
| Jun-Jul | 0.47 | *0.01* | 0 | 0.71 | 0 |
|  |  |  |  |  |  |
| Aug | 0.15 | 0.19 | 0 | 0.49 | 0.01 |
|  |  |  |  |  |  |
| Phytoplankton |  |  |  |  |  |
|  |  |  |  |  |  |
| May | 0.06 | 0.34 | 0 | 0.89 | 0.09 |
|  |  |  |  |  |  |
| Jun-Jul | 0.08 | 0.25 | 0.14 | 0.12 | 0 |
|  |  |  |  |  |  |
| Aug | 0 | 0.51 | 0 | 0.75 | 0.06 |
|  |  |  |  |  |  |

[E‌│S], variation explained by pure environmental variables, contribution of S removed.

[S‌│E], variation explained by pure spatial variables, contribution of E removed.

[E‌+S], variation explained by both environment and space (spatially structured environment). Significance of this fraction cannot be tested.

Significant probability values in italics.

**Table S7.** Effects of lake type, defined by the presence and life-history form of alewives (*Alosa pseudoharengus*), on species diversity, species richness, and beta diversity of zooplankton and phytoplankton, as analyzed with repeated measures ANOVA.

|  |  |  | | |
| --- | --- | --- | --- | --- |
| Response variable | d.f. | *F* | *P* | Contrasts |
|  |  |  |  |  |
|  |  |  |  |  |
| Zooplankton |  |  |  |  |
|  |  |  |  |  |
| Species diversity |  |  |  |  |
|  |  |  |  |  |
| Lake type | 2, 9 | 1.70 | 0.235 |  |
|  |  |  |  |  |
| Time | 2, 18 | 0.54 | 0.590 |  |
|  |  |  |  |  |
| Time x lake type | 4, 18 | 0.80 | 0.543 |  |
|  |  |  |  |  |
| Species richness |  |  |  |  |
|  |  |  |  |  |
| Lake type | 2, 9 | 8.26 | *0.009* | (A, N↑**) |
|  |  |  |  |  |
| Time | 2, 18 | 2.49 | 0.111 |  |
|  |  |  |  |  |
| Time x lake type | 4, 18 | 1.68 | 0.199 |  |
|  |  |  |  |  |
| Beta diversity |  |  |  |  |
|  |  |  |  |  |
| Lake type | 2, 9 | 5.54 | *0.016* | (A↑, L*), (L, N↑*) |
|  |  |  |  |  |
| Time | 2, 18 | 0.24 | 0.788 |  |
|  |  |  |  |  |
| Time x lake type | 4, 18 | 0.99 | 0.426 |  |
|  |  |  |  |  |
| Phytoplankton |  |  |  |  |
|  |  |  |  |  |
| Genus diversity |  |  |  |  |
|  |  |  |  |  |
| Lake type | 2, 9 | 17.32 | *0.001* | (A, L↑**), (L↑, N***) |
|  |  |  |  |  |
| Time | 2, 18 | 0.32 | 0.728 |  |
|  |  |  |  |  |
| Time x lake type | 4, 18 | 0.88 | 0.498 |  |
|  |  |  |  |  |
| Genus richness |  |  |  |  |
|  |  |  |  |  |
| Lake type | 2, 9 | 1.87 seven | 0.209 |  |
|  |  |  |  |  |
| Time | 2, 18 | 2.19 | 0.841 |  |
|  |  |  |  |  |
| Time x lake type | 4, 18 | 0.61 | 0.662 |  |
|  |  |  |  |  |

**Table S7.**

**Table S7. continued**

|  |  |  | | |
| --- | --- | --- | --- | --- |
| Response variable | d.f. | *F* | *P* | Contrasts |
|  |  |  |  |  |
|  |  |  |  |  |
| Phytoplankton (continued) |  |  |  |  |
|  |  |  |  |  |
| Beta diversity |  |  |  |  |
|  |  |  |  |  |
| Lake type | 2, 9 | 2.22 | 0.143 |  |
|  |  |  |  |  |
| Time | 2, 18 | 1.14 | 0.332 |  |
|  |  |  |  |  |
| Time x lake type | 4, 18 | 2.11 | 0.104 |  |
|  |  |  |  |  |
|  |  |  |  |  |
|  |  |  |  |  |

Significant probability values appear in italics. Post-hoc pairwise comparisons (Tukey’s HSD) are reported for significant main effects. Abbreviations for post-hoc contrasts: A = anadromous alewife, L = landlocked alewife, N = no alewife. Contrasts significance levels: *, *P* < 0.05; **, *P* < 0.01; ***, *P* < 0.001; ↑, greater diversity value.

**Table S8.** Effects of lake type, defined by the presence and life-history form of alewives (*Alosa pseudoharengus*), on species diversity, species richness, and beta diversity of zooplankton and phytoplankton functional groups, as analyzed with repeated measures ANOVA.

**Table S8**.

|  |  |  | | |
| --- | --- | --- | --- | --- |
| Response variable | d.f. | *F* | *P* | Contrasts |
|  |  |  |  |  |
|  |  |  |  |  |
| Large zooplankton |  |  |  |  |
|  |  |  |  |  |
| Species diversity |  |  |  |  |
|  |  |  |  |  |
| Lake type | 2, 9 | 10.72 | *0.004* | (A, N↑**), (L, N↑*) |
|  |  |  |  |  |
| Time | 2, 18 | 0.30 point | 0.750 |  |
|  |  |  |  |  |
| Time x lake type | 4, 18 | 0.30 | 0.879 |  |
|  |  |  |  |  |
| Species richness |  |  |  |  |
|  |  |  |  |  |
| Lake type | 2, 9 | 42.49 | *<0.001* | (A, N↑***), (L, N↑***) |
|  |  |  |  |  |
| Time | 2, 18 | 1.56 | 0.237 seven |  |
|  |  |  |  |  |
| Time x lake type | 4, 18 | 1.20 | 0.345 |  |
|  |  |  |  |  |
| Beta diversity |  |  |  |  |
|  |  |  |  |  |
| Lake type | 2, 9 | 2.94 | 0.084 |  |
|  |  |  |  |  |
| Time | 2, 18 | 0.10 | 0.904 |  |
|  |  |  |  |  |
| Time x lake type | 4, 18 | 2.17 | 0.097 |  |
|  |  |  |  |  |
| Small zooplankton |  |  |  |  |
|  |  |  |  |  |
| Species diversity |  |  |  |  |
|  |  |  |  |  |
| Lake type | 2, 9 | 0.45 | 0.654 |  |
|  |  |  |  |  |
| Time | 2, 18 | 1.47 | 0.257 |  |
|  |  |  |  |  |
| Time x lake type | 4, 18 | 0.49 | 0.741 |  |
|  |  |  |  |  |
| Species richness |  |  |  |  |
|  |  |  |  |  |
| Lake type | 2, 9 | 1.42 | 0.292 |  |
|  |  |  |  |  |
| Time‡ | 2, 18 | 2.06 | 0.177 |  |
|  |  |  |  |  |
| Time x lake type‡ | 4, 18 | 1.35 | 0.303 |  |
|  |  |  |  |  |

|  |  |  | | |
| --- | --- | --- | --- | --- |
| Response variable | d.f. | *F* | *P* | Contrasts |
|  |  |  |  |  |
|  |  |  |  |  |
| Small zooplankton (continued) |  |  |  |  |
|  |  |  |  |  |
| Beta diversity |  |  |  |  |
|  |  |  |  |  |
| Lake type | 2, 9 | 4.73 | *0.026* | (A↑, L*), (L, N↑†) |
|  |  |  |  |  |
| Time | 2, 18 | 1.79 | 0.184 |  |
|  |  |  |  |  |
| Time x lake type | 4, 18 | 0.33 | 0.854 |  |
|  |  |  |  |  |
| Edible phytoplankton |  |  |  |  |
|  |  |  |  |  |
| Genus diversity |  |  |  |  |
|  |  |  |  |  |
| Lake type | 2, 9 | 3.37 | 0.080 |  |
|  |  |  |  |  |
| Time | 2, 18 | 5.14 | *0.017* |  |
|  |  |  |  |  |
| Time x lake type | 4, 18 | 1.33 | 0.297 |  |
|  |  |  |  |  |
| Genus richness |  |  |  |  |
|  |  |  |  |  |
| Lake type | 2, 9 | 0.67 | 0.533 |  |
|  |  |  |  |  |
| Time | 2, 18 | 0.36 | 0.701 |  |
|  |  |  |  |  |
| Time x lake type | 4, 18 | 0.16 | 0.954 |  |
|  |  |  |  |  |
| Beta diversity |  |  |  |  |
|  |  |  |  |  |
| Lake type | 2, 9 | 3.03 | 0.078 |  |
|  |  |  |  |  |
| Time | 2, 18 | 0.02 | 0.985 |  |
|  |  |  |  |  |
| Time x lake type | 4, 18 | 1.10 | 0.376 |  |
|  |  |  |  |  |
|  |  |  |  |  |
|  |  |  |  |  |

**Table S8. continued**

**Table S8. continued**

|  |  |  | | |
| --- | --- | --- | --- | --- |
| Response variable | d.f. | *F* | *P* | Contrasts |
|  |  |  |  |  |
|  |  |  |  |  |
| Inedible phytoplankton |  |  |  |  |
|  |  |  |  |  |
| Genus diversity |  |  |  |  |
|  |  |  |  |  |
| Lake type | 2, 9 | 2.28 | 0.158 |  |
|  |  |  |  |  |
| Time | 2, 18 | 3.17 | 0.066 |  |
|  |  |  |  |  |
| Time x lake type | 4, 18 | 1.63 | 0.209 |  |
|  |  |  |  |  |
| Genus richness |  |  |  |  |
|  |  |  |  |  |
| Lake type | 2, 9 | 2.45 | 0.141 |  |
|  |  |  |  |  |
| Time | 2, 18 | 0.60 | 0.561 |  |
|  |  |  |  |  |
| Time x lake type | 4, 18 | 1.05 | 0.408 |  |
|  |  |  |  |  |
| Beta diversity |  |  |  |  |
|  |  |  |  |  |
| Lake type | 2, 9 | 2.49 | 0.116 |  |
|  |  |  |  |  |
| Time | 2, 18 | 2.60 | 0.091 |  |
|  |  |  |  |  |
| Time x lake type | 4, 18 | 5.15 | *0.003* |  |
|  |  |  |  |  |
|  |  |  |  |  |

‡ Greenhouse-Geisser modified probability values

Significant probability values appear in italics. Post-hoc pairwise comparisons (Tukey’s HSD) are reported for significant main effects. Abbreviations for post-hoc contrasts: A = anadromous alewife, L = landlocked alewife, N = no alewife. Contrasts significance levels: †, marginally significant *P* = 0.06; *, *P* < 0.05; **, *P* < 0.01; ***, *P* < 0.001; ↑, greater diversity value.

**Table S9.** Species contributing to the similarity of zooplankton communities by lake type, defined by the presence and life-history form of alewives (*Alosa pseudoharengus*), during the May, June-July, and August 2009 sampling periods. The percentage contribution of each species to the compositional similarity of lakes within each lake type, and body size of each species (large-bodied, length ≥0.8 mm; small-bodied, length <0.8 mm), are noted. Absence of percentage data indicates that the species does not significantly contribute to compositional similarity among lakes in the lake type.

|  |  |  |  |  |
| --- | --- | --- | --- | --- |
| Species | Size | Anadromous | Landlocked | No alewife |
|  |  |  |  |  |
|  |  |  |  |  |
| May |  |  |  |  |
|  |  |  |  |  |
| *Acanthocyclops vernalis* | Small | 8.37 | 19.02 | 4.32 |
|  |  |  |  |  |
| *Bosmina longirostris* | Small | 23.67 | 34.37 | 5.08 |
|  |  |  |  |  |
| *Chydorus sphaericus* | Small | -- | 7.11 | -- |
|  |  |  |  |  |
| *Daphnia ambigua* | Large | 46.10 | -- | 3.19 |
|  |  |  |  |  |
| *Daphnia catawba* | Large | -- | -- | 3.41 |
|  |  |  |  |  |
| *Diacyclops thomasi* | Small | -- | 28.08 | 2.76 |
|  |  |  |  |  |
| *Diaphanosoma* | Small | -- | -- | 2.69 |
|  |  |  |  |  |
| *Eubosmina* | Small | 15.26 | -- | -- |
|  |  |  |  |  |
| *Mesocyclops edax* | Large | -- | -- | 8.32 |
|  |  |  |  |  |
| *Skistodiaptomus* | Large | -- | 5.90 | 62.06 |
|  |  |  |  |  |
| Jun-Jul |  |  |  |  |
|  |  |  |  |  |
| *Acanthocyclops vernalis* | Small | 4.21 | -- | -- |
|  |  |  |  |  |
| *Bosmina longirostris* | Small | 79.92 | 39.91 | -- |
|  |  |  |  |  |
| *Ceriodaphnia lacustris* | Small | -- | 12.07 | -- |
|  |  |  |  |  |
| *Diaphanosoma* | Small | -- | 5.91 | 5.56 |
|  |  |  |  |  |
| *Mesocyclops edax* | Large | 6.13 | 9.51 | 47.99 |
|  |  |  |  |  |

**Table S9. continued**

|  |  |  |  |  |
| --- | --- | --- | --- | --- |
| Species | Size | Anadromous | Landlocked | No alewife |
|  |  |  |  |  |
|  |  |  |  |  |
| Jun-Jul (continued) |  |  |  |  |
|  |  |  |  |  |
| *Skistodiaptomus* | Large | -- | 19.78 | 20.14 |
|  |  |  |  |  |
| *Tropocyclops extensis* | Small | -- | 5.39 | -- |
|  |  |  |  |  |
| Aug |  |  |  |  |
|  |  |  |  |  |
| *Acanthocyclops vernalis* | Small | -- | 8.84 | -- |
|  |  |  |  |  |
| *Bosmina longirostris* | Small | 68.29 | 40.92 | -- |
|  |  |  |  |  |
| *Ceriodaphnia lacustris* | Small | -- | 18.50 | -- |
|  |  |  |  |  |
| *Daphnia catawba* | Large | -- | -- | 5.93 |
|  |  |  |  |  |
| *Diaphanosoma* | Small | -- | -- | 20.09 |
|  |  |  |  |  |
| *Eubosmina* | Small | 14.56 | -- | -- |
|  |  |  |  |  |
| *Mesocyclops edax* | Large | 7.63 | 12.33 | 24.31 |
|  |  |  |  |  |
| *Skistodiaptomus* | Large | -- | 10.42 | 39.63 |
|  |  |  |  |  |
| *Tropocyclops extensis* | Small | -- | -- | 3.84 |
|  |  |  |  |  |

**Table S10.** Genera contributing to the similarity of phytoplankton communities by lake type, defined by the presence and life-history form of alewives (*Alosa pseudoharengus*), during the August 2009 sampling period. The percentage contribution of each genus to the compositional similarity of lakes within each lake type, and the edibility of the genus to zooplankton (edible, ≤35μm; inedible, >35 μm), are noted. Absence of percentage data indicates that the genus does not significantly contribute to compositional similarity among lakes in the lake type.

|  |  |  |  |  |
| --- | --- | --- | --- | --- |
| Genus | Edibility | Anadromous | Landlocked | No alewife |
|  |  |  |  |  |
|  |  |  |  |  |
|  |  |  |  |  |
|  |  |  |  |  |
| *Ceratium* | Inedible | -- | 15.62 | 17.99 |
|  |  |  |  |  |
| *Chlamydomonas* | Edible | 16.46 | 2.49 | -- |
|  |  |  |  |  |
| *Chlorella* | Edible | 4.53 | 4.82 | 6.98 |
|  |  |  |  |  |
| *Cryptomonas* | Edible | 9.45 | 14.37 | 29.67 |
|  |  |  |  |  |
| *Cyclotella* | Edible | 24.26 | -- | 6.74 |
|  |  |  |  |  |
| *Dinobryon* | Inedible | -- | 9.81 | -- |
|  |  |  |  |  |
| *Euglena* | Inedible | -- | 7.61 | 10.02 |
|  |  |  |  |  |
| *Gymnodinium* | Edible | 28.17 | 12.51 | 4.46 |
|  |  |  |  |  |
| *Oscillatoria* | Inedible | -- | -- | 6.36 |
|  |  |  |  |  |
| *Peridinium* | Inedible | 10.71 | 21.76 | -- |
|  |  |  |  |  |
| *Trachelomonas* | Edible | -- | 2.15 | 9.69 |
|  |  |  |  |  |
|  |  |  |  |  |
|  |  |  |  |  |
